# Editorial: Psychodidactic Variables and Academic Performance in Physical Education

**DOI:** 10.3389/fpsyg.2021.652702

**Published:** 2021-03-30

**Authors:** Antonio Granero-Gallegos, Raúl Baños, Antonio Baena-Extremera

**Affiliations:** ^1^Faculty of Education Sciences, University of Almeria, Almeria, Spain; ^2^Health Research Center, University of Almeria, Almeria, Spain; ^3^Faculty of Sciences for Physical Activity and Sport (INEF), Polytechnic University of Madrid, Madrid, Spain; ^4^Faculty of Education Sciences, University of Granada, Granada, Spain

**Keywords:** physical activity, physical education, psychodidactics, academic performance, education

Research in recent years has demonstrated that various psychological and didactic variables can significantly affect students, even affecting academic performance. Among these variables are techniques such as creating a positive class climate, encouraging intrinsic motivation to study school subjects, providing autonomy to the student in undertaking their homework, using various teaching-learning models, and even promoting an active lifestyle. It is also important to point out that scientific literature on this subject has shown that many of these variables can be approached from the point of view of the teacher or that of the student and that there is a close relationship between the development and evolution of the students' lives and their future school performance.

The articles collected in this Research Topic cover a huge variety of approaches (both qualitative and quantitative), including countries (such as Germany, Italy, Mexico, Romania, and Spain, etc.), and different types of studies (e.g., empirical research, research-action, training, validation of instruments, etc.).

This wealth of innovative material is offered with the aim of not only deepening knowledge on the subject but also offering practical ways to improve the psychological and didactic variables that could affect students.

Articles were submitted between November 2019 and June 2020, and involving 67 authors. The special issue includes 14 papers, discussed here according to the chronological order of article publication.

Granero-Gallegos et al. use multilevel regression models to understand differences in school satisfaction, disruptive behaviors, and teaching competencies according to the gender of students. They analyze school satisfaction and disruptive student behaviors based on perceived teaching competence. The study highlights that boys showed higher levels of negative behaviors than girls. In addition, it highlights that physical education (PE) teacher competence influences disruptive behaviors in the classroom, and that this is also related to school satisfaction.

Chiva-Bartoll et al. use a quasi-experimental design to analyze the effects of a service learning program on subjective happiness, prosocial behavior, and professional learning perceptions of Physical Education Teacher Education students, to examine correlations among these variables. This investigation provides educational researchers with valuable information to better understand how service-learning influences the training of Physical Education Teacher Education students.

Zamarripa et al. asses the psychometric properties, structure, and factorial invariance by gender of the adaptation of Basic Psychological Need Satisfaction and Frustration Scale to the PE context in Mexico. The authors demonstrate that this scale can be used to measure the satisfaction and/or frustration of the basic psychological needs of students in PE class and to assess differences between boys and girls.

Salvador-García et al. analyze the impact of content and language integrated learning (CLIL) on PE lessons through qualitative and quantitative approaches. The authors found that levels of moderate to vigorous physical activity are higher in the experimental group (CLIL) than in the control group, a result that clarifies the divergent viewpoints of the interviewees. However, they did not find differences related to social relationships.

Manzano-Sánchez et al. evaluated a program based on Hellison's Teaching Personal and Social Responsibility Model using qualitative methodologies. This study concludes that the model can be applied to all participants in the curriculum and is adaptable to any content and type of student body. The authors suggest the application of the model by all teachers involved in the same school year and including the participation of students' families.

Through a longitudinal study, Gentile et al. assess the effects of a PE program, elaborated within the Enriched Sports Activity Program (ESA Program) of an Erasmus + Project, on executive functions, namely, visuospatial working memory, inhibitory control, cognitive flexibility, and task switching. The authors conclude that the introduction of a sports program enriched with cognitive stimuli has beneficial effects on the working memory and cognitive flexibility of children.

Segura-Robles et al. analyze the effects of a flipped and gamified program on autonomy, competence, relatedness, satisfaction/enjoyment, intrinsic and extrinsic motivation, and boredom of students in PE. This study was carried out through experimental and pre-post design. The results indicated that autonomy students' satisfaction, enjoyment, and intrinsic motivation have improved based on the interaction with gamification and flipped learning.

Hortigüela-Alcalá et al. assess to what extent future PE teachers can apply the training they received at university in the classroom, deepening their fears, insecurities, and problems when carrying it out. A qualitative methodology was used and the results showed how the future teachers did not see their expectations of success fulfilled, encountering resistance from both students and teachers in PE.

Fernández-Espínola et al. check a model that analyzed the prediction of the satisfaction of the need for autonomy, competence, relatedness, and novelty, as well as the motivation experienced in PE on the intention to be physically active. A questionnaire was administered to 1665 PE students and path analysis were performed to conclude the importance of satisfying all the basic psychological needs (including novelty) and give special emphasis to the need for competence, since it predicts autonomous motivation and the intention to be physically active outside of the educational context.

Gea-García et al. analyze the mediating role of integrated regulation on the relationship between physical activity and physical fitness in children and adolescents. The authors conclude that mediation of the integrated regulation could be decisive in predicting and explaining the relationship between the practice of physical activity and physical fitness at these ages, highlighted its importance for greater adherence to the practice.

Claver et al. determine a predictive model of disciplined behaviors and academic performance in Physical Education students using achievement goal theory and self-determination theory as the theoretical framework. The results highlight the importance of the task-oriented motivational climate and the mediating role of basic psychological needs and autonomous motivation to generate these positive student outcomes (discipline and academic performance) in PE.

Crisol-Moya et al. focus their research on active methodologies in higher education. They analyze the opinions about active methodologies among university professors and students, describing perception and opinion of the modes of organization, methodological focuses, and evaluation systems that define the teaching-learning process. The results show that professors and students think they are making progress toward a learning-centered model, and that implementation of active methodologies implies new functions in their teaching practice.

Vergara-Torres et al. test a model of multilevel mediation that examines the relationships between the perception of corrective feedback with its degree of acceptance (perceived legitimacy) at the team level and the subjective vitality of students at the individual level, mediated by the satisfaction of three psychological needs, in the context of physical education. Based on self-determination theory, this research concludes that students who perceive their basic psychological needs to be met experience an increase their subjective vitality.

Mosoi et al. conduct a cross-sectional study to test the differences between organized physical activity and non-organized physical activity in an after-school program. A multivariate analysis of variance was used to conclude that not participating in an organized physical activity program results in a reduced level of physical mobility and consequently is associated with maladaptive social and psychological outcomes.

In conclusion, these 14 papers contribute to current scientific knowledge related to psychodidactic variables and academic performance in the field of PE. This Research Topic responds to the concerns of the different agents that intervene in the teaching-learning process through different stages, from elementary school to university

## Author Contributions

All authors contributed to the article and approved the submitted version.

## Conflict of Interest

The authors declare that the research was conducted in the absence of any commercial or financial relationships that could be construed as a potential conflict of interest. The handling editor declared a shared affiliation with one of the authors AG-G at time of review.

